# Supplementary material for: User perspectives on the Molbio Truenat platform and tuberculosis assays for decentralised testing in Mozambique and Tanzania
Source: BMJ Glob Health. 2026 May 11;11(5):e019902. doi: 10.1136/bmjgh-2025-019902 (PMC13182370; doi:10.1136/bmjgh-2025-019902)
Supplement: online supplemental file 1 [file bmjgh-11-5-s001.pdf]

COREQ (COnsolidated criteria for REporting Qualitative research) Checklist

A checklist of items that should be included in reports of qualitative research. You must report the page number in your manuscript where you consider each of the items listed in this checklist. If you have not included this information, either revise your manuscript accordingly before submitting or note N/A.

Topic	Item No.	Guide Questions/Description	Reported on Page No.
**Domain 1: Research team and reflexivity**			
Personal characteristics			
Interviewer/facilitator	1	Which author/s conducted the interview or focus group?	Page 6 and reflexivity statement file
Credentials	2	What were the researcher's credentials? E.g. PhD, MD	
Occupation	3	What was their occupation at the time of the study?	
Gender	4	Was the researcher male or female?	
Experience and training	5	What experience or training did the researcher have?	
Relationship with participants			
Relationship established	6	Was a relationship established prior to study commencement?	No personal relationship existed prior to data collection. Interviewers introduced themselves and addressed participant questions after being referred by local researchers.
Participant knowledge of the interviewer	7	What did the participants know about the researcher? e.g. personal goals, reasons for doing the research	Interviewers introduced themselves, their institutional affiliation, and the study objectives, including their interest in TB diagnostics and the purpose of the research.
Interviewer characteristics	8	What characteristics were reported about the inter viewer/facilitator? e.g. Bias, assumptions, reasons and interests in the research topic	Interviewers were nationals of the countries where the study was conducted, with experience and research interest in TB diagnostics. More details are listed in the reflexivity statement file.
**Domain 2: Study design**			
Theoretical framework			
Methodological orientation and Theory	9	What methodological orientation was stated to underpin the study? e.g. grounded theory, discourse analysis, ethnography, phenomenology, content analysis	Page 6, "Data Entry and Analysis"

Participant selection			
Sampling	10	How were participants selected? e.g. purposive, convenience, consecutive, snowball	Page 5, "Study Settings and Participants"
Method of approach	11	How were participants approached? e.g. face-to-face, telephone, mail, email	Page 6, "Data Collection Methods"
Sample size	12	How many participants were in the study?	Page 5, 7, tables 1 and 2
Non-participation	13	How many people refused to participate or dropped out? Reasons?	
Setting			
Setting of data collection	14	Where was the data collected? e.g. home, clinic, workplace	Page 6, "Data collection methods"
Presence of nonparticipants	15	Was anyone else present besides the participants and researchers?	
Description of sample	16	What are the important characteristics of the sample? e.g. demographic data, date	Tables 1 and 2
**Topic**	**Item No.**	**Guide Questions/Description**	**Reported on Page No.**
Data collection			
Interview guide	17	Were questions, prompts, guides provided by the authors? Was it pilot tested?	Page 6, "Data collection methods"
Repeat interviews	18	Were repeat inter views carried out? If yes, how many?	N/A (no repeated interviews)
Audio/visual recording	19	Did the research use audio or visual recording to collect the data?	Page 6, "Data entry and analysis"
Field notes	20	Were field notes made during and/or after the inter view or focus group?	Page 6, "Data entry and analysis"
Duration	21	What was the duration of the inter views or focus group?	Page 6, "Data collection methods"
Data saturation	22	Was data saturation discussed?	Page 6, "Data entry and analysis"
Transcripts returned	23	Were transcripts returned to participants for comment and/or correction?	
**Domain 3: analysis and findings**			
Data analysis			
Number of data coders	24	How many data coders coded the data?	The initial coding was conducted by one researcher (MDMC), with iterative discussions involving local investigators to refine the coding tree.
Description of the coding tree	25	Did authors provide a description of the coding tree?	Page 6, "Data entry and analysis". The coding tree was based on the updated CFIR, covering domains such as innovation factors, inner setting, individuals (recipients, HCPs, DMs), and outer setting.

Derivation of themes	26	Were themes identified in advance or derived from the data?	Page 6, "Data entry and analysis"
Software	27	What software, if applicable, was used to manage the data?	Page 6, "Data entry and analysis"
Participant checking	28	Did participants provide feedback on the findings?	Page 19
Reporting			
Quotations presented	29	Were participant quotations presented to illustrate the themes/findings?<br>Was each quotation identified? e.g. participant number	Pages 8 – 16, Result section.
Data and findings consistent	30	Was there consistency between the data presented and the findings?	Pages 8 – 16, Result section.
Clarity of major themes	31	Were major themes clearly presented in the findings?	Pages 8 – 16, Result section.<br>Table 3
Clarity of minor themes	32	Is there a description of diverse cases or discussion of minor themes?	Pages 8 – 16, Result section.

Developed from: Tong A, Sainsbury P, Craig J. Consolidated criteria for reporting qualitative research (COREQ): a 32-item checklist for interviews and focus groups. *International Journal for Quality in Health Care*. 2007. Volume 19, Number 6: pp. 349 – 357
