# Supplementary material for: User perspectives on the Molbio Truenat platform and tuberculosis assays for decentralised testing in Mozambique and Tanzania
Source: BMJ Glob Health. 2026 May 11;11(5):e019902. doi: 10.1136/bmjgh-2025-019902 (PMC13182370; doi:10.1136/bmjgh-2025-019902)
Supplement: online supplemental file 2 [file bmjgh-11-5-s002.pdf]

Reflexivity statement

1. How does this study address local research and policy priorities?

This study is aligned with the priorities of national tuberculosis program in Mozambique and Tanzania, particularly the need to improve access to timely and accurate TB diagnosis in decentralized and resource-limited settings. By evaluating the implementation of the Truenat platform and gathering perspectives from people undergoing TB testing, healthcare providers, and decision-makers, the study provides context-specific evidence to inform scale-up strategies. The focus on user experience, context-specific barriers, and operational feasibility provides critical information to local efforts to strengthen diagnostic capacity and aligns with broader global goals to end TB.

2. How were local researchers involved in study design?

Local researchers were closely involved in all phases of the study, including its design, adaptation of data collection tools, and piloting of instruments to ensure cultural and contextual relevance. They led the data collection and contributed to the interpretation of findings and manuscript preparation, supporting a collaborative and context-informed approach. The local teams—comprising both women and men from diverse backgrounds in medicine, public health, and social sciences—brought varying levels of experience in qualitative research. While the Tanzanian teams had prior experience, for some members of the Mozambique teams this was their first qualitative study. All field teams received training workshops covering the study's objectives, data collection procedures, and interviewing techniques, and had prior training in Good Clinical Practice and research ethics through their involvement in the broader TB-CAPT CORE study.

3. How has funding been used to support the local research team?

Study funding was used to support operational activities, including participant recruitment, training sessions, and salaries. These resources ensured the effective conduct of the study and contributed to capacity building within the research team.

4. How are research staff who conducted data collection acknowledged?

Researchers involved in data collection were actively involved throughout the study, contributing to instrument refinement, fieldwork, and interpretation of findings. Their meaningful engagement in the research process is recognized through co-authorship, in accordance with established authorship criteria.

5. Do all members of the research partnership have access to study data?

All partnership institutions have equal access to the study data through secure platforms. While the first author led the primary analysis, all collaborators are able to use the dataset to explore additional research questions, supporting transparency, shared ownership, and equitable collaboration across the partnership.

6. How was data used to develop analytical skills within the partnership?

Analytical capacity was strengthened through ongoing collaboration, including regular meetings where team members reviewed, discussed and iteratively improved the coding framework, as well as interpreted emerging data together. Input from co-investigators was

actively incorporated to ensure contextual relevance. This collaborative and iterative process supported skill development in qualitative analysis and understanding of the data.

7. How have research partners collaborated in interpreting study data?

Interpretation of study data was a joint effort, involving continuous dialogue through virtual meetings, collaborative reviews, and written exchanges. During data collection, debriefing sessions helped refine methods and ensure data quality. In the later stages, partners engaged in interpretation of the findings, with particular attention to country-specific contexts and differences.

8. How were research partners supported to develop writing skills?

Writing skills were supported through a collaborative drafting process. The first author, a PhD student, led the initial draft and facilitated structured discussions with local teams to incorporate their input. This approach allowed co-authors to engage meaningfully and gain experience with the writing process.

9. How will research products be shared to address local needs?

The publication will be made openly accessible and shared as a pre-print to maximize dissemination. Locally, the findings from this and the larger clinical trial will be translated into relevant languages and shared through community engagement sessions, presentations or briefs tailored to local stakeholders (policymakers, health providers and others) to inform decision-making.

10. How is the leadership, contribution, and ownership of this work by LMIC researchers recognized within the authorship?

Leadership and contribution from LMIC researchers are reflected in the authorship, with several authors based in the countries where the study was conducted. The first author, a PhD student originally from an LMIC, played a central role in leading the research and manuscript preparation.

11. How have early-career researchers across the partnership been included within the authorship team?

Early-career researchers, including research assistants, were actively engaged throughout the research process and met the criteria for authorship. Their contributions, which span from data collection to analysis and interpretation, are reflected in the manuscript and explicitly acknowledged in the author contribution statements.

12. How has gender balance been addressed within the authorship?

The authorship team comprises 8 women and 11 men, with women holding both the first and senior author positions.

13. How has the project contributed to training of LMIC researchers?

The project supported LMIC researcher training through workshops on qualitative data collection and analysis, provision of NVivo software licenses, and funded participation in international conferences (e.g., the Union World TB Conference). A PhD student from an LMIC

is completing their dissertation as part of this project, contributing to their academic and professional development.

14. How has the project contributed to improvements in local infrastructure?

While the project did not directly improve physical infrastructure, it strengthened research capacity and further strengthened sustainable research partnerships that will benefit future studies. These partnerships create a foundation for continued collaborative research and capacity building in both countries. The broader consortium will contribute to the Truenat implementation, strengthening local capacities for TB testing.

15. What safeguarding procedures were used to protect local study participants and researchers?

The study implemented comprehensive safeguarding procedures. Prior to study enrollment, all study staff were provided training (or refresher) for good clinical practices. All procedures were approved by ethics committees in Germany, Mozambique, and Tanzania, according to research best practices. Written standard operating protocols were implemented and training on these protocols were provided to all study staff to safeguard both participants and researchers, with regular monitoring[1] to ensure compliance.

References

1. Leukes VN, Hella J, Sabi I, Cossa M, Khosa C, Erkosar B, et al. Study protocol: a pragmatic, cluster-randomized controlled trial to evaluate the effect of implementation of the Truenat platform/MTB assays at primary health care clinics in Mozambique and Tanzania (TB-CAPT CORE). BMC Infect Dis. 2024;24(1):107. Epub 20240119. doi: 10.1186/s12879-023-08876-8. PubMed PMID: 38243223; PubMed Central PMCID: PMCPMC10797907.
